# Evaluation of alternative respiratory syndromes for specific syndromic surveillance of influenza and respiratory syncytial virus: a time series analysis

**DOI:** 10.1186/1471-2334-9-190

**Published:** 2009-11-29

**Authors:** Suzanne K Schindeler, David J Muscatello, Mark J Ferson, Kris D Rogers, Paul Grant, Tim Churches

**Affiliations:** 1 Centre for Epidemiology and Research, New South Wales Department of Health, North Sydney, Australia; 2 South Eastern Sydney and Illawarra Public Health Unit, Randwick, New South Wales, Australia; 3 School of Public Health & Community Medicine, University of New South Wales, Sydney, Australia

## Abstract

**Background:**

Syndromic surveillance is increasingly being evaluated for its potential for early warning of increased disease activity in the population. However, interpretation is hampered by the difficulty of attributing a causative pathogen. We described the temporal relationship between laboratory counts of influenza and respiratory syncytial virus (RSV) detection and alternative groupings of Emergency Department (ED) respiratory diagnoses.

**Methods:**

ED and laboratory data were obtained for the south-eastern area of Sydney, NSW for the period 1 June 2001 - 1 December 2006. Counts of ED visits and laboratory confirmed positive RSV and influenza cases were aggregated by week. Semi-parametric generalized additive models (GAM) were used to determine the association between the incidence of RSV and influenza and the incidence of respiratory syndrome ED presentations while controlling for temporal confounders.

**Results:**

For every additional RSV laboratory count, ED diagnoses of bronchiolitis increased by 3.1% (95%CI: 2.7%-3.5%) in the same week. For every additional influenza laboratory count, ED diagnoses of influenza-like illness increased by 4.7% (95%CI: 4.2%-5.2%) one week earlier.

**Conclusion:**

In this study, large increases in ED diagnoses of bronchiolitis and influenza-like illness were independent and proxy indicators for RSV and influenza activity, respectively.

## Background

Syndromic surveillance is increasingly being used for monitoring disease activity because of its potential for early detection of outbreaks and epidemics [1-6], and its potentially widespread coverage of target populations. However, interpretation of surveillance signals is often hampered by the difficulty of implicating a causative pathogen. There is a need to understand whether and how syndromic surveillance can distinguish between specific pathogens circulating in the population.

In temperate climate zones, emergency department visits for respiratory conditions such as bronchiolitis, influenza-like illness, and pneumonia have been found to display a distinctly seasonal pattern, with ED visits peaking in the winter months [7,8]. Previous studies have found that influenza virus and respiratory syncytial virus (RSV) explain most of the variation in presentations of respiratory syndromes to EDs [9,10,7], but these studies did not determine whether syndromic surveillance could distinguish between these viruses.

RSV is the most common cause of lower respiratory tract infection in infants and children worldwide and often manifests as bronchiolitis and pneumonia [11,12]. Almost all children have been infected with RSV by two years of age and re-infection throughout life is common. In adults, RSV is increasingly recognized as an important cause of serious respiratory disease in the elderly and immuno-compromised individuals [11]. In younger, otherwise healthy adults, RSV may have a clinical presentation similar to influenza [13].

Apart from causing typical influenza syndromes, influenza viruses have a well-established relationship with pneumonia morbidity and mortality [14] and can also be a cause of bronchiolitis [15] in younger children. There is strong evidence that RSV and influenza co-circulate [14] and co-infection is possible [16].

Another important consideration for syndromic surveillance is whether it can offer earlier warning of disease activity than surveillance of specific pathogens. Our previous work found at least a 3 day advantage of monitoring daily counts of emergency department diagnoses of influenza compared with laboratory surveillance of influenza [8]. Wijngaard et al [9] found between 0 and 5 weeks advantage for alternative respiratory illness syndromes compared with influenza, and between 3 weeks disadvantage and 2 weeks advantage for the same syndromes against laboratory-confirmed RSV. However, the respiratory syndromes were non-specific and did not discriminate between those pathogens.

No studies, to our knowledge, have investigated whether surveillance of ED diagnoses of specific respiratory syndromes can distinguish between different causative pathogens circulating in the population. Hence, this time series study aimed to determine how RSV and influenza virus activity in the population affect alternative ED-based respiratory syndrome definitions in terms of the degree of association and timing. Understanding this relationship between ED syndromes and underlying viral activity may help in interpreting increases in syndrome activity observed in syndromic surveillance.

## Methods

### Setting and data sources

RSV is not a notifiable/reportable condition in New South Wales (NSW), Australia. However, we obtained RSV laboratory data from public hospital laboratories participating in the Eastern Sydney Laboratory Surveillance Program, which covers the south-eastern area of Sydney. Influenza is required to be notified by laboratories to the NSW Department of Health [17] and was thus obtained from the NSW Notifiable Diseases Database. Records were selected if the notifying public health unit was within the south-eastern area of Sydney. ED data was obtained from the NSW Emergency Department Data Collection [18] derived from the six public hospitals in the same geographic area. The ED data collection is drawn from data entered in information systems in NSW EDs used by ED personnel for patient management. The longest time period of available data common to all datasets was 1^st ^June 2001 - 1^st ^December 2006.

### Syndrome definitions

Syndrome definitions were based on those used in existing ED-based syndromic surveillance in NSW [4]. The system defines syndromes using provisional primary diagnoses selected in patient management information systems used in EDs. These information systems automatically record the corresponding International Classification of Diseases (ICD) Version 9 or 10 code, depending on the information system used. "Bronchiolitis syndrome" was defined as ED presentations assigned a diagnosis of bronchiolitis (ICD-9-CM code 466.1, or ICD-10-AM code J21). "Pneumonia syndrome" was defined as a diagnosis of pneumonia (ICD-9-CM codes 480-486, or ICD-10-AM codes J12-J18). "Influenza-like syndrome" was defined as a diagnosis of influenza (ICD-9-CM code 487, or ICD-10-AM codes J10 and J11). "All acute respiratory infection" syndrome included diagnoses of whooping cough, acute upper respiratory infections, influenza and pneumonia, other acute lower respiratory infections, and cough (ICD -9-CM: 033, 460-466, 480-487, 786.2; ICD-10-AM: A37, J00-J22, R05). "All respiratory syndrome" consisted of the respiratory conditions mentioned above as well as asthma, chronic obstructive pulmonary disease, respiratory distress or arrest, other breathing difficulties, and respiratory conditions during the perinatal period (ICD-9-CM: 033, 460-519, 768-770, 799.1, 786.09, 786.2; ICD-10-AM: A37, J00-J99, P20-P28, R09.2, R06.8, R05).

### Analysis

Counts of ED visits and laboratory confirmed positive RSV and influenza cases were aggregated by week. Week of ED visit was used for the ED time series and the week of specimen collection was used for the laboratory series. Semi-parametric generalized additive models (GAM) were used to determine the association between the incidence of RSV and influenza and the incidence of respiratory syndrome ED presentations. GAMs extend traditional GLMs by replacing linear predictors of the form:

η = ∑_j_β_j_χ_j _with η = ∑_j_f_j_(χ_j_) where f_j_(χ_j_) can be nonparametric smooth functions [19,20], thus incorporating the flexibility of nonparametric regression while still retaining the interpretability of GLMs [21]. Alternative methods, such as Poisson regression (without the use of a spline) but with the inclusion of a covariate to control for seasonality have been used in other studies [22], but were unable to remove autocorrelation in the residuals in our data. The non-parametric flexibility of GAMs has resulted in their widespread use in time-series studies to adjust for the nonlinear confounding effects of seasonality and trend [19,23-27].

The counts of ED visits were assumed to follow a Poisson distribution. Five models were constructed - one for each ED syndrome: bronchiolitis, pneumonia, influenza-like, all acute respiratory infections, and all respiratory visits. The outcome in each model was the time-series of weekly ED visits for the syndrome.

In each model, laboratory counts of RSV and influenza were included as predictors, as well as a non-parametric spline term for time (in weeks) to control for seasonal and secular trends. These factors need to be controlled because they produce autocorrelation (serial dependence) in the model residuals. Like our previous study of just influenza, we used natural cubic smoothing splines to control for autocorrelation and trend [8]. Failure to control for these factors can lead to incorrect inference in time series analysis. Because this study was using weekly rather than daily time increments, we used 4 degrees of freedom per year in the splines as this was sufficient for removing autocorrelation in the residuals for most of the time series we examined. This effectively removed variation occurring over more than a quarterly period and was a good balance between removing too much trend and leaving too little short-term variation in the time series. Autocorrelation was assessed by visual inspection of autocorrelation plots and p-values using the ARIMA procedure in SAS version 9.1.

Where the spline was insufficient to remove autocorrelation from the model residuals an autoregressive term (the previous week's ED syndrome count) was included in the model to remove this residual autocorrelation. This was necessary for the all acute respiratory infections syndrome and the all respiratory syndrome models. Not removing this residual autocorrelation would result in a failure of the modelling assumption of independence in the residuals and thus to incorrect inference.

The modelling was completed in two stages. The first stage was to determine the time lag in weeks that produced the strongest association between each single laboratory time series and the ED syndrome. The lag which produced the strongest association was taken to be the relative risk which was furthest from unity. The second stage was to include the lag producing the strongest association in a final model that included both RSV and influenza as explanatory variables. The lag could be different for each of RSV and influenza. Laboratory time series were lagged in single weeks from -4 to +4 weeks to allow a reasonable window for each virus to plausibly influence ED visits. The final models for each ED syndrome outcome were of the form:(1)

where Y_t _denotes the weekly count of ED visits for the syndrome, β_1 _denotes the log relative risk of ED visits associated with a one-unit increase in laboratory confirmed positive RSV infections, β_2 _denotes the log relative risk of ED visits associated with a one-unit increase in laboratory confirmed positive influenza infections, and S(time) is the smoothing spline for time (in weeks). LagRSV and lagInfluenza represent the lags at which the strongest association occurred for the individual laboratory series.

Analysis was performed using the GAM procedure in SAS version 9.1. This study used de-identified epidemiological information and therefore ethical approval was not required.

## Results

### Descriptive statistics

**Figure 1:**
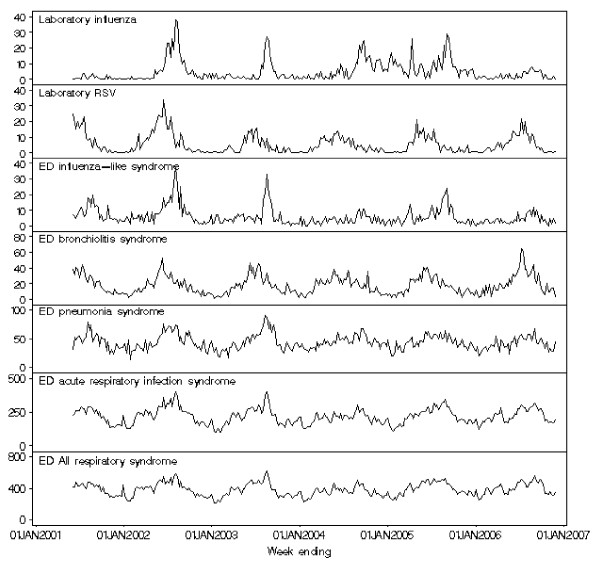
**Weekly counts of Emergency Department (ED) syndromes and laboratory syndromes: 1 June 2001 to 1 December 2006**.

Visual inspection of the time-series of counts revealed similar timing of the seasonal peaks for the ED bronchiolitis syndrome and laboratory RSV, but the peaks for laboratory influenza occurred several weeks later (Figure 1). The seasonal peaks for ED pneumonia syndrome occurred after the peaks for laboratory RSV, but shortly before the peaks for laboratory influenza. The seasonal peaks for ED influenza-like syndrome occurred after the peaks for laboratory RSV, and at a similar time to the peaks of laboratory influenza. Compared with the all acute respiratory infection syndrome, and the all respiratory syndrome, the peaks for laboratory RSV occurred earlier, while the peaks for laboratory influenza occurred later.

The age distribution for the data is shown in Table 1. As expected, the majority of RSV laboratory diagnoses occurred in infants aged <1 year (65.1%), whereas influenza laboratory diagnoses were fairly evenly distributed amongst adults aged 17-34 years (20.4%), 35-64 years (25.8%), and 65+ years (24.4%). The bronchiolitis ED syndrome mostly occurred in infants aged <1 year (86.7%), the pneumonia syndrome mostly occurred in adults aged 65+ years (43.2%), and the influenza-like syndrome mostly occurred in adults aged 17-34 years (40.8%). The all acute respiratory infections syndrome, and the all respiratory syndrome, mainly occurred in children aged 1-4 years (30.1% and 23.2% respectively). The proportions of ED visits admitted to hospital wards were 35.6% for the bronchiolitis, 5.8% for the influenza-like, 24.4% for the all acute respiratory infection, 34.6% for the all respiratory, and 73.8% for the pneumonia ED syndromes, respectively.

**Table 1:** Number (and percent total) per age group, for laboratory and Emergency Department (ED) syndrome data^1^.

Age group	Laboratory influenza	Laboratory RSV	ED bronchiolitis	ED pneumonia	ED influenza-like	ED acute respiratory infections	ED all respiratory
<1 yr	68 (4.8)	1021 (65.1)	4570 (86.7)	405 (3.2)	36 (2.1)	11531 (18.4)	13094 (11.9)
1-4 yrs	157 (11.1)	443 (28.3)	632 (12.0)	1961 (15.6)	103 (6.1)	18820 (30.1)	25509 (23.2)
5-16 yrs	189 (13.4)	38 (2.4)	21 (0.4)	1356 (10.8)	265 (15.8)	10976 (17.5)	17077 (15.5)
17-34 yrs	289 (20.4)	19 (1.2)	11 (0.2)	956 (7.6)	685 (40.8)	7517 (12.0)	15341 (13.9)
35-64 yrs	367 (25.9)	41 (2.6)	16 (0.3)	2446 (19.5)	489 (29.1)	6466 (10.3)	16168 (14.7)
65+	345 (24.4)	6 (0.4)	23 (0.4)	5419 (43.2)	102 (6.1)	7278 (11.6)	22797 (20.7)
Total	1415	1568	5273	12543	1680	62588	109986

1. Source: ED data sourced from the NSW Emergency Department Data Collection. Laboratory Influenza data sourced from the NSW Notifiable Diseases Database. Laboratory RSV data sourced from the Eastern Sydney Laboratory Surveillance Program, NSW. Time period for all data: 1 June 2001-1 December 2006.

Influenza A virus accounted for the majority of positive influenza laboratory cases (77.7%). Influenza B accounted for 19.8% of the cases, while 2.5% were positive for both influenza A and B.

### Preliminary GAMs to determine lags with largest effects

The results of the preliminary analysis to establish the lags for the final models are given in Table 2. Relative risks refer to changes in ED visits resulting from every additional positive laboratory report for influenza and RSV. ED bronchiolitis syndrome was most associated with RSV laboratory counts in the same week (lag 0 week) (RR 1.031, 95%CI: 1.027-1.035), and with influenza laboratory counts four weeks in the future (lag -4 weeks) (RR: 1.007, 95%CI: 1.003-1.011). ED pneumonia syndrome was most associated with RSV counts four weeks in the past (lag + 4 weeks) (RR: 1.015, 95%CI: 1.012-1.018), and with influenza laboratory counts one week in the future (lag -1 week) (RR: 1.011, 95%CI: 1.008-1.013). ED influenza-like syndrome was most associated with RSV counts one week in the past (lag 1 week) (RR: 0.983, 95%CI: 0.976-0.990), and with influenza laboratory counts one week in the future (lag -1 week) (RR: 1.047, 95%CI: 1.042-1.052). The all acute respiratory infections and all respiratory syndromes were most associated with RSV laboratory counts occurring two weeks in the past (RR: 1.008, 95%CI: 1.006-1.009; and RR: 1.006, 95%CI: 1.005-1.007 respectively), and influenza laboratory counts occurring one week in the future (RR: 1.012, 95%CI: 1.011-1.013; and RR: 1.009, 95%CI: 1.008-1.010 respectively).

**Table 2:** Change in Emergency Department (ED) syndrome counts (relative risk (RR) and 95% CI) associated with a 1 unit increase in weekly laboratory counts, lagged by up to ± 4 weeks: Univariate analysis.

ED syndrome outcome	Lag^1 ^(weeks)	RR RSV (95%CI)	RSV P-value	RR influenza (95%CI)	Influenza P-value
Bronchiolitis	-4	1.009 (1.004-1.013)	0.0001	**1.007 (1.003-1.011)**	< 0.0001
	-3	1.015 (1.011-1.019)	< 0.0001	1.003 (1.000-1.007)	0.075
	-2	1.021 (1.017-1.026)	< 0.0001	0.998 (0.994-1.001)	0.221
	-1	1.025 (1.020-1.029)	< 0.0001	0.999(0.995-1.003)	0.658
	0	**1.031 (1.027-1.035)**	< 0.0001	0.999 (0.995-1.003)	0.586
	1	1.020 (1.016-1.024)	< 0.0001	0.995 (0.990-0.999)	0.009
	2	1.018 (1.014-1.022)	< 0.0001	1.000 (0.996-1.005)	0.815
	3	1.015 (1.011-1.019)	< 0.0001	1.000 (0.995-1.004)	0.838
	4	1.015 (1.011-1.019)	< 0.0001	0.998 (0.994-1.003)	0.461

Pneumonia	-4	0.998 (0.995-1.001)	0.1088	1.004 (1.002-1.007)	0.0005
	-3	0.998 (0.995-1.001)	0.1611	1.007 (1.005-1.009)	< 0.0001
	-2	1.001 (0.998-1.004)	0.4498	1.010 (1.008-1.012)	< 0.0001
	-1	1.000 (0.997-1.003)	0.8307	**1.011 (1.008-1.013)**	< 0.0001
	0	1.004 (1.001-1.007)	0.0043	1.009 (1.007-1.012)	< 0.0001
	1	1.005 (1.002-1.008)	0.0008	1.007 (1.005-1.009)	< 0.0001
	2	1.011 (1.008-1.014)	< 0.0001	1.003 (1.000-1.005)	0.0303
	3	1.011 (1.008-1.013)	< 0.0001	1.004 (1.002-1.007)	0.0011
	4	**1.015 (1.012-1.018)**	< 0.0001	0.999 (0.996-1.001)	0.3567

Influenza - like	-4	1.009 (1.001-1.016)	0.0285	0.994 (0.988-1.000)	0.0383
	-3	0.988 (0.981-0.996)	0.0031	1.011 (1.006-1.017)	< 0.0001
	-2	0.984 (0.976-0.991)	< 0.0001	1.029 (1.024-1.034)	< 0.0001
	-1	0.984 (0.977-0.992)	< 0.0001	**1.047 (1.042-1.052**)	< 0.0001
	0	0.991 (0.984-0.998)	0.0129	1.041 (1.036-1.046)	< 0.0001
	1	**0.983 (0.976-0.990)**	< 0.0001	1.025 (1.020-1.031)	< 0.0001
	2	1.006 (0.999-1.012)	0.1129	1.025 (1.020-1.031)	< 0.0001
	3	1.002 (0.995-1.009)	0.5857	1.014 (1.008-1.020)	< 0.0001
	4	0.996 (0.989-1.002)	0.2172	0.999 (0.993-1.005)	0.655

All acute respiratory infections	-4	0.998 (0.997-0.999)	< 0.0001	1.003 (1.002-1.005)	< 0.0001
	-3	0.997 (0.996-0.998)	< 0.0001	1.006 (1.005-1.007)	< 0.0001
	-2	0.999 (0.998-1.001)	0.2516	1.008 (1.007-1.009)	< 0.0001
	-1	1.002 (1.001-1.003)	0.0032	**1.012 (1.011-1.013)**	< 0.0001
	0	1.004 (1.003-1.005)	< 0.0001	1.010 (1.009-1.011)	< 0.0001
	1	1.002 (1.001-1.003)	0.0053	1.007 (1.006-1.008)	< 0.0001
	2	**1.008 (1.006-1.009)**	< 0.0001	1.005 (1.004-1.006)	< 0.0001
	3	1.007 (1.006-1.008)	< 0.0001	1.003 (1.002-1.004)	< 0.0001
	4	1.006 (1.005-1.008)	< 0.0001	0.999 (0.998-1.000)	0.0876

All respiratory	-4	1.000 (0.999-1.001)	0.5467	1.003 (1.002-1.004)	< 0.0001
	-3	0.999 (0.998-1.000)	0.1499	1.005 (1.004-1.006)	< 0.0001
	-2	1.001 (1.000-1.002)	0.2258	1.007 (1.006-1.008)	< 0.0001
	-1	1.003 (1.002-1.004)	< 0.0001	**1.009 (1.008-1.010)**	< 0.0001
	0	1.005 (1.004-1.005)	< 0.0001	1.007 (1.006-1.008)	< 0.0001
	1	1.002 (1.001-1.003)	0.001	1.004 (1.004-1.005)	< 0.0001
	2	**1.006 (1.005-1.007)**	< 0.0001	1.003 (1.002-1.004)	< 0.0001
	3	1.006 (1.005-1.006)	< 0.0001	1.001 (1.000-1.002)	0.0198
	4	1.005 (1.004-1.006)	< 0.0001	0.998 (0.997-0.999)	< 0.0001

^1^A negative lag number refers to virus counts occurring after ED visits, while a positive lag number refers to virus counts occurring before ED visits.

### Final GAMs

The results of the final models that included both RSV and influenza are presented in Table 3. After controlling for long term trend and seasonality, for every additional RSV laboratory count, the bronchiolitis ED syndrome increased by 3.1% (95%CI: 2.7%-3.5%) in the same week, the pneumonia syndrome increased by 1.4% (95%CI: 1.1%-1.7%) four weeks in the future, the influenza-like syndrome decreased by 2.0% (95%CI: 1.3%-2.8%) one week in the future, the all acute respiratory infection syndrome increased by 0.6% (95%CI: 0.5%-0.7%) two weeks in the future, and the all respiratory syndrome increased by 0.4% (95%CI: 0.3%-0.5%) two weeks in the future.

**Table 3:** Change in ED visits (Relative risk and 95%CI) associated with a 1 unit increase in weekly laboratory counts: final models.

ED syndrome outcome	Covariates (lag, weeks)	RR (95%CI)	P-value
Bronchiolitis	RSV (0)	1.031 (1.027-1.035)	< 0.0001
	Influenza (-4)	1.006 (1.003-1.010)	0.001

Pneumonia	RSV (+4)	1.014 (1.011-1.017)	< 0.0001
	Influenza (-1)	1.010 (1.007-1.012)	< 0.0001

Influenza - like	RSV (+1)	0.980 (0.972-0.987)	< 0.0001
	Influenza (-1)	1.047 (1.042-1.052)	< 0.0001

All respiratory	RSV (2)	1.004 (1.003-1.005)	< 0.0001
	Influenza (-1)	1.005 (1.004-1.006)	< 0.0001
	All respiratory ED visits 1 week prior	1.001 (1.001-1.001)	< 0.0001

All acute respiratory infections	RSV (2)	1.006 (1.005-1.007)	< 0.0001
	Influenza (-1)	1.006 (1.005-1.007)	< 0.0001
	All respiratory ED visits 1 week prior	1.002 (1.002-1.002)	< 0.0001

^1^Only lags between -4 and +4 weeks were considered in this study.

After controlling for long term trend and seasonality, for every additional influenza laboratory count, the bronchiolitis syndrome increased by 0.6% (95%CI: 0.3%-1.0%) 4 weeks in the past, the pneumonia syndrome increased by 1.0% (95%CI: 0.7%-1.2%) one week in the past, the influenza-like syndrome increased by 4.7% (95%CI: 4.2%-5.2%) one week in the past, the all acute respiratory infection syndrome increased by 0.6% (95%CI: 0.5%-0.7%) one week in the past, and the all respiratory syndrome increased by 0.5% (95%CI: 0.4%-0.6%) one week in the past.

**Figure 2:**
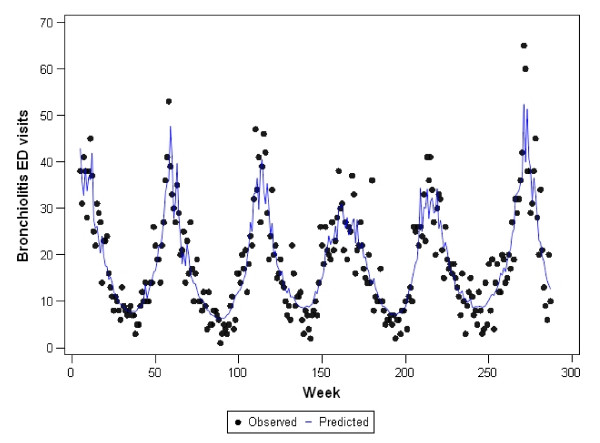
**Observed versus predicted values for bronchiolitis ED visits by week: 1 June 2001 to 1 December 2006**.

**Figure 3:**
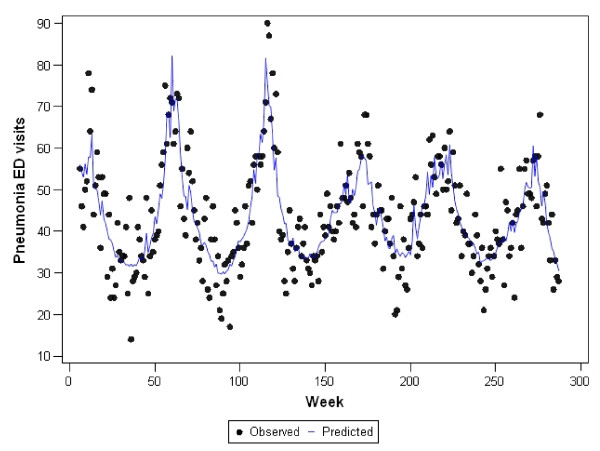
**Observed versus predicted values for pneumonia ED visits by week: 1 June 2001 to 1 December 2006**.

**Figure 4:**
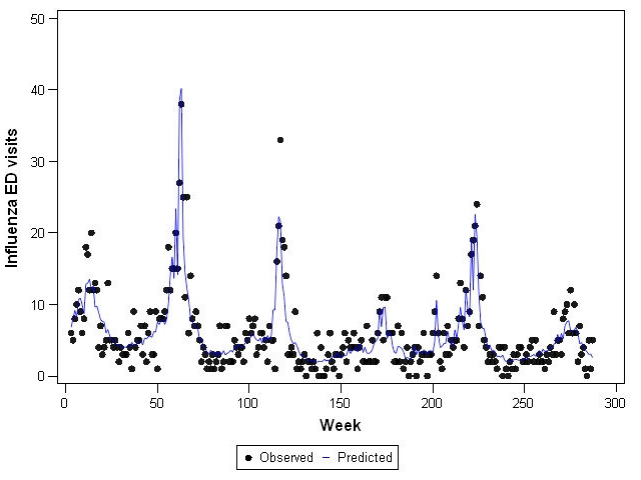
**Observed versus predicted values for influenza ED visits by week: 1 June 2001 to 1 December 2006**.

**Figure 5:**
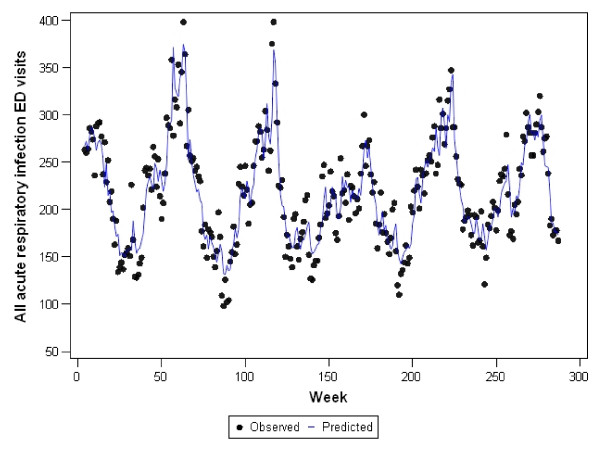
**Observed versus predicted values for all acute respiratory infections ED visits by week: 1 June 2001 to 1 December 2006**.

**Figure 6:**
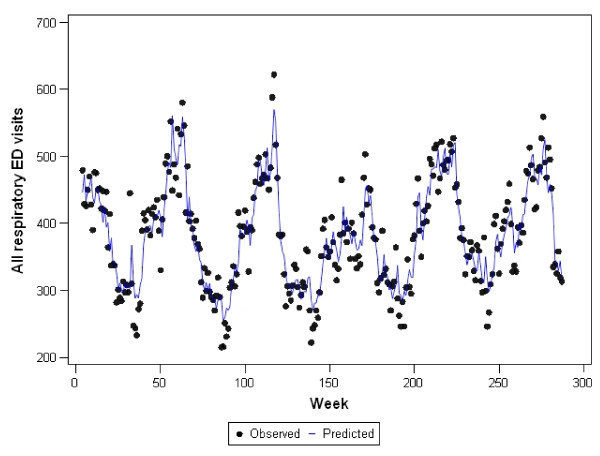
**Observed versus predicted values for all respiratory ED visits by week: 1 June 2001 to 1 December 2006**.

Graphs representing the observed counts for ED visits versus the fitted (predicted) values for each of the final models are shown in Figures 2, 3, 4, 5, and 6. These figures reveal good fit for each of the final models.

## Discussion

By fitting time-series models which control for both longer-term and seasonal effects, and which account for the inherent auto-correlation in the data, we found a large, significant, and independent association between ED presentations for influenza and positive laboratory tests for influenza viruses. We also found a large, significant and independent association between ED presentations for bronchiolitis, and RSV laboratory counts. Thus our results confirm the value of monitoring these more specific syndromes in discriminating between influenza and RSV activity in the population.

While the relative increases in ED visits in the bronchiolitis and influenza-like syndromes associated with a unit increase in laboratory-identified RSV and influenza virus were of the order of 3% and 5%, respectively, a small increase in laboratory identified virus could actually represent a very large increase in population levels of illness. This is because only a small proportion of people exposed to the virus and infected are likely to attend an ED and an even smaller proportion are likely to be tested for a virus.

The other, smaller associations found in this study are more difficult to interpret. In the period we studied, RSV laboratory counts peaked before influenza laboratory counts, and RSV was associated with ED diagnoses of pneumonia several weeks later. Hence, it is possible that RSV infection may increase subsequent susceptibility to influenza infection or pneumonia, although other studies are needed to investigate this further.

The relatively small associations found between laboratory RSV and influenza counts, and the all acute respiratory infection and all respiratory syndromes indicate that there are many other factors driving the increase in these ED visits which have not been accounted for in the models for these two syndrome outcomes. These other factors may include additional circulating respiratory pathogens, environmental contributors including temperature, or holidays. The inclusion of the previous week's ED visits in the GAM models for both of these syndromes was required to remove autocorrelation in the residuals, and represents the contribution of these unmeasured factors.

For influenza, our findings were consistent with our previous work using a similar date range and state-wide data, and a different, but sound, statistical method [8]. Our findings were also broadly consistent with those of Wijngaard et al [9] who also controlled for autocorrelation in their regression model. They found a 1-2 week advantage for hospitalisations diagnosed with any acute respiratory illness over laboratory-identified influenza. For RSV, they found that the hospitalisations increased in the same week as laboratory-identified RSV.

The data sources used in this study may have some limitations. Since RSV data was only available for the south-eastern area of Sydney, the scope of our study was limited to this region. Therefore, due to small numbers, analysis by age group for instance was not possible. Another limitation is that ED provisional diagnoses were selected by ED medical, nursing or clerical staff in the course of their work, and selection of codes may vary between staff and hospitals. Limitations for the ED data and laboratory influenza data are discussed further in [8].

Our decision to use GAMs in this study was based on the flexibility they provide through the use of nonparametric regression. The problem with using a parametric approach, such as sinusoidal terms to control for seasonality, is that this assumes that the seasonal peak is the same height and occurs at the same time each year [28]. It can clearly be seen from the time-series graphs (Fig. 1) that this is not the case in our data, and is probably not the case in these time series generally.

## Conclusion

In conclusion, syndromic surveillance of ED visits diagnosed with influenza or bronchiolitis can give a reasonable assurance that influenza and RSV, respectively, are circulating in the population, and can be used to discriminate between them. This finding is particularly useful in our state, where RSV infection is not a reportable disease and near real-time ED surveillance is a reality [4].

## Competing interests

The authors declare that they have no competing interests.

## Authors' contributions

SS designed and performed the analysis and drafted the manuscript. DM conceived of the study, participated in its design and coordination, assisted in the interpretation of the results, and helped to draft the manuscript. MF was involved in the design and interpretation of the study and assisted in its interpretation. KR and PG were involved in the initial literature review and early analyses. TC contributed to the design of the study and assisted in its interpretation. All authors read and approved the final manuscript.

## Pre-publication history

The pre-publication history for this paper can be accessed here:

http://www.biomedcentral.com/1471-2334/9/190/prepub
